# PARP1 Co-Regulates EP300–BRG1-Dependent Transcription of Genes Involved in Breast Cancer Cell Proliferation and DNA Repair

**DOI:** 10.3390/cancers11101539

**Published:** 2019-10-11

**Authors:** Maciej Sobczak, Andrew R. Pitt, Corinne M. Spickett, Agnieszka Robaszkiewicz

**Affiliations:** 1 Department of General Biophysics, Institute of Biophysics, Faculty of Biology and Environmental Protection, University of Lodz, Pomorska 141/143, 90-236 Lodz, Poland; maciej.sobczak@unilodz.eu; 2 School of Life & Health Sciences, Aston University, Aston Triangle, Birmingham B4 7ET, UK; a.r.pitt@aston.ac.uk (A.R.P.); c.m.spickett@aston.ac.uk (C.M.S.)

**Keywords:** poly-ADP-ribose polymerase 1 (PARP1), brahma-related gene 1 (BRG1), histone acetyltransferase p300 (EP300), gene transcription, cancer cell

## Abstract

BRG1, an active subunit of the SWI/SNF chromatin-remodeling complex, enables the EP300-dependent transcription of proliferation and DNA repair genes from their E2F/CpG-driven promoters in breast cancer cells. In the current study, we show that BRG1–EP300 complexes are accompanied by poly-ADP-ribose polymerase 1 (PARP1), which emerges as the functional component of the promoter-bound multiprotein units that are capable of controlling gene expression. This enzyme is co-distributed with BRG1 at highly acetylated promoters of genes such as *CDK4*, *LIG1,* or *NEIL3*, which are responsible for cancer cell growth and the removal of DNA damage. ADP-ribosylation is necessary to maintain active transcription, since it ensures an open chromatin structure that allows high acetylation and low histone density. PARP1-mediated modification of BRG1 and EP300 does not affect the association of enzymes with gene promoters; however, it does activate EP300, which acetylates nucleosomes, leading to their eviction by BRG1, thus allowing mRNA synthesis. Although PARP1 was found at BRG1 positive/H3K27ac negative promoters of highly expressed genes in a transformed breast cancer cell line, its transcriptional activity was limited to genes simultaneously controlled by BRG1 and EP300, indicating that the ADP-ribosylation of EP300 plays a dominant role in the regulation of BRG1–EP300-driven transcription. In conclusion, PARP1 directs the transcription of some proliferation and DNA repair genes in breast cancer cells by the ADP-ribosylation of EP300, thereby causing its activation and marking nucleosomes for displacement by BRG1. PARP1 in rapidly dividing cells facilitates the expression of genes that confer a cancer cell phenotype. Our study shows a new mechanism that links PARP1 with the removal of DNA damage in breast cancer cells via the regulation of BRG1–EP300-dependent transcription of genes involved in DNA repair pathways.

## 1. Introduction

The pharmacological effects of inhibitors of poly-ADP-ribose polymerases (PARPs) in anticancer therapies are attributed to impairing DNA damage removal, since PARP1 plays a crucial role in the recruitment of repair machinery, mainly in an ADP-ribosylation-dependent manner [1,2]. Lesion recognition followed by the recruitment of poly-ADP-ribose polymerase 1 (PARP1) to sites of DNA damage and ADP-ribosylation of its automodification domain are prerequisites for the binding of XRCC1, POLB, LIG3, or ALC1, which are involved in base excision repair (BER), single-strand break repair (SSBR), and nucleotide excision repair (NER) [3,4]. However, PARP1 also facilitates alternative and conventional non-homologous end-joining (NHEJ) as well as homologous recombination (HR), therefore helping to protect genome integrity and preventing destabilization of the genome resulting from double-strand breaks [5,6,7]. According to the “access–repair–restore” model, nucleic acid repair is preceded by chromatin reorganization, since DNA lesions are curtained by DNA-associated proteins [8]. Thus, local chromatin rearrangements are required to allow the assembly of the multiprotein machinery that removes lesions. Recent findings identified a link between PARP1 activity, nucleosome density, and the efficiency of some repair pathways, including HR [9]. In the study referred to, auto-ADP-ribosylated PARP1 serves as an indispensable anchor that provides a platform at the damage site for the functional interaction between the nucleosome-evicting brahma-related gene 1 (BRG1; the SWI/SNF chromatin-remodeling enzyme) and the NAD-dependent deacetylase sirtuin-1 (SIRT1), which activates BRG1 by erasing lysine acetylation, thus promoting DNA end-joining. BRG1 interacts with the poly-ADP-ribose polymer through its ATPase domain rather than the N- or C-terminal tails, and is recruited to genomic regions enriched in PARP1. A previous paper reported the interaction of BRG1 with PARP1 and other histone-remodeling enzymes at the genomic level, where nucleosome-evicting ATPase cooperated with PARP1 and histone deacetylases (HDACs: HDAC2 and HDAC9) at the promoters of *α-MHC* and *β-MHC*, thereby preventing cardiac differentiation and maintaining the proliferation potential of the precursors [10]. However, the molecular mechanism that drives PARP1/BRG1-dependent up- or down-regulation of gene transcription has not yet been identified. The suggestion that PARP1 enables the binding of EP300 to the promoters of cell cycle-dependent genes in proliferating cells in an ADP-ribosylation-independent manner focused our attention on the possible role of PAR-synthesizing enzymes in the transcriptional regulation of genes controlled by BRG–EP300–HDAC1 complexes [11]. Our recent discoveries regarding the above-mentioned chromatin-remodeling units showed that these enzymes control the transcription of proliferation and DNA repair genes in two considerably different breast cancer cell lines, MCF7 and MDA-MB-231, which differ in terms of their expressed hormone and HER2 receptors [12]. The nucleosomes of E2F/CpG-driven promoters in the two studied gene groups were acetylated by histone acetyltransferase p300 (EP300), causing them to be marked for BRG1-mediated eviction, and enabling paused RNA polymerase II to become active, leading to active gene transcription. This mechanism operates only in proliferating cells, which are also characterized by a high abundance of PARP1. This is because the *PARP1* promoter is controlled by BRG1–EP300–HDAC1 complexes and is repressed with respect to the growth arrest seen in the great majority of normal primary cells [11].

Based on these premises, we aimed to discover whether PARP1 co-regulates BRG1–EP300-dependent transcription, and if PARP1 can be considered an active component of such multiprotein complexes in the studied breast cancer cell lines. We also aimed to uncover the molecular mechanism that links PARP1 with BRG1-dependent transcription and verify possible PARP1 selectivity toward functionally related genes.

## 2. Results

### 2.1. PARP1 Physically Interacts with SWI/SNF in Breast Cancer Cells

Data from three biological replicates run in duplicate for the PARP immunoprecipitates (IP) and two biological replicates in duplicate for the control IP were analyzed. A total of 76 interacting proteins were identified that fulfilled the selection criteria (confidence scores >50, fold change >2 and *p*-values < 0.05; the full list of PARP1-interacting proteins and associated data are shown in Table S1). Since PARP1 has been previously identified as a cofactor of the transcriptional machinery that cooperates with histone-remodeling enzymes and transcription factors [13,14,15,16], we focused on the identification of proteins that physically interacted with PARP1 within the cell nucleus, and initially assessed the interaction data for new, previously unidentified chromatin-associated proteins that could be involved in the regulation of gene expression in a PARP1-dependent fashion. The analysis of PARP1 co-immunoprecipitated proteins by mass spectrometry identified DNA-bound subunits of RNA polymerase and mediator complexes, as well as subunits of chromatin-remodeling complexes, such as Tip60, p400 (EP400), and SWI/SNF (ARID1A, SMARCC1, BRG1; Table S1), which were significantly overrepresented in PARP1 versus IgG pull-downs. Interestingly, brahma (BRM), the other ATPase subunit in SWI/SNF, was not detected among the significant number of interacting proteins identified in PARP1 immunoprecipitates in any of three biological replicates from peptide identification in Mascot. This finding may suggest that PARP1 cooperates only with BRG1-based SWI/SNF complexes. However, to conclude on PARP1–BRM physical and functional interaction, further examination is needed. 

**Figure 1:**
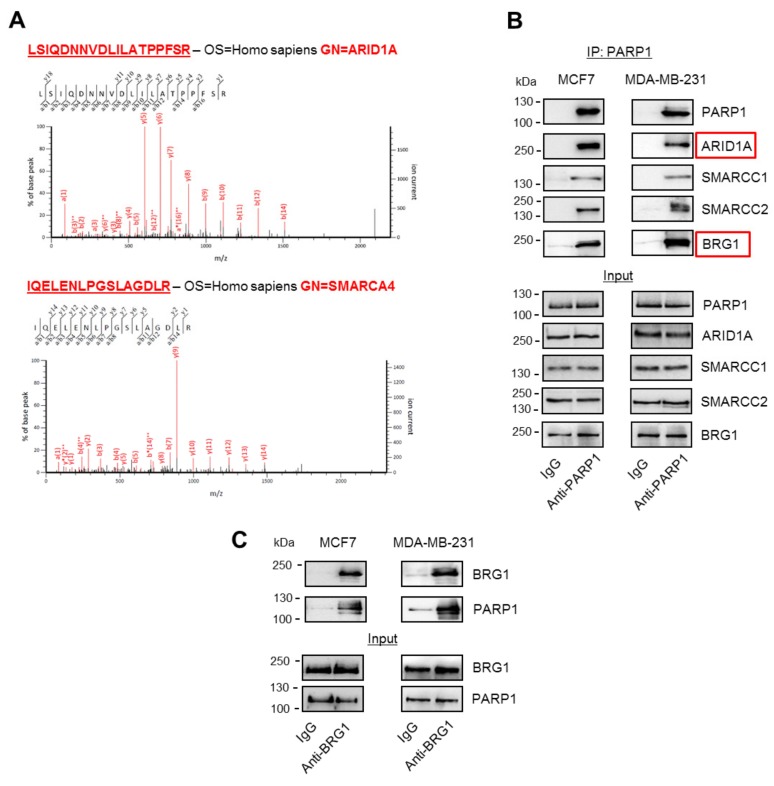
Poly-ADP-ribose polymerase 1 (PARP1) physically interacts with the brahma-related gene 1 (BRG1)-based SWI/SNF chromatin-remodeling complex. (**A**) Exemplar mass spectrometry (MSMS; representative peptides from three biological replicates visualized in Mascot) data for the identification of ARID1 and SMARCA4 as PARP1 interactors using PARP1 immunoenrichment. (**B**) PARP1 interaction with ARID1A, SMARCC1, SMARCC2, and SMARCA4/BRG1 was confirmed by PARP1 pull-down and protein detection by Western blotting in cell lysates of two breast cancer cell lines, MCF7 and MDA-MB-231. IgG served as an isotypic control. (**C**) PARP1 was also identified in BRG1 immunoprecipitates. Western blotting images show representative images of three biological, fully reproducible replicates.

Among other histone writers, erasers, and readers, we also found HDAC1, which was recently reported by us to be a constitutive component the BRG1–EP300–HDAC1 complex and assembles at the cell cycle-driven gene promoters of, for example, DNA repair genes in human macrophages and breast cancer cells [11,12]. To verify that formaldehyde fixation of the nuclei did not lead to false positive readouts with mass spectrometry and whether PARP1 is a bona fide member of the SWI/SNF complexes, PARP1 was immunoprecipitated from intact cells, and pull-downs were tested using Western blotting for the presence of SWI/SNF components previously detected by mass spectrometry in fixed nuclei. Western blotting of PARP1 co-immunoprecipitated proteins confirmed the direct interaction of PARP1 with ARID1A and BRG1, but also with other subunits of SWI/SNF, such as SMARCC1 and SMARCC2, in the studied breast cancer cell lines (Figure 1B). Similarly, PARP1 was detected in BRG1 pull-downs (Figure 1C).

### 2.2. PARP1 Is Co-Distributed with BRG1 at the Actively Transcribed Gene Promoters

To confirm the possible role of PARP1 in the regulation of SWI/SNF-dependent transcription, we first tested whether PARP1 occurrence in the genome of breast cancer cells was accompanied by BRG1. The residual signal from PARP1 and BRG1 is randomly distributed across genomes, and may reflect the antibody specificity (or lack thereof) or experimental challenges rather than (or in addition to) true protein occurrence. However, the local enrichment of these proteins can be observed, and PARP1 and BRG1-rich regions can be identified by Model-based Analysis of ChIP-Seq data (MACS). As described in the Methods section, for these proteins, we set a *p*-value cutoff for peak detection at 10-3 with three levels of regions around the peak region (1, 5, and 10 kbp) assessed.

**Figure 2:**
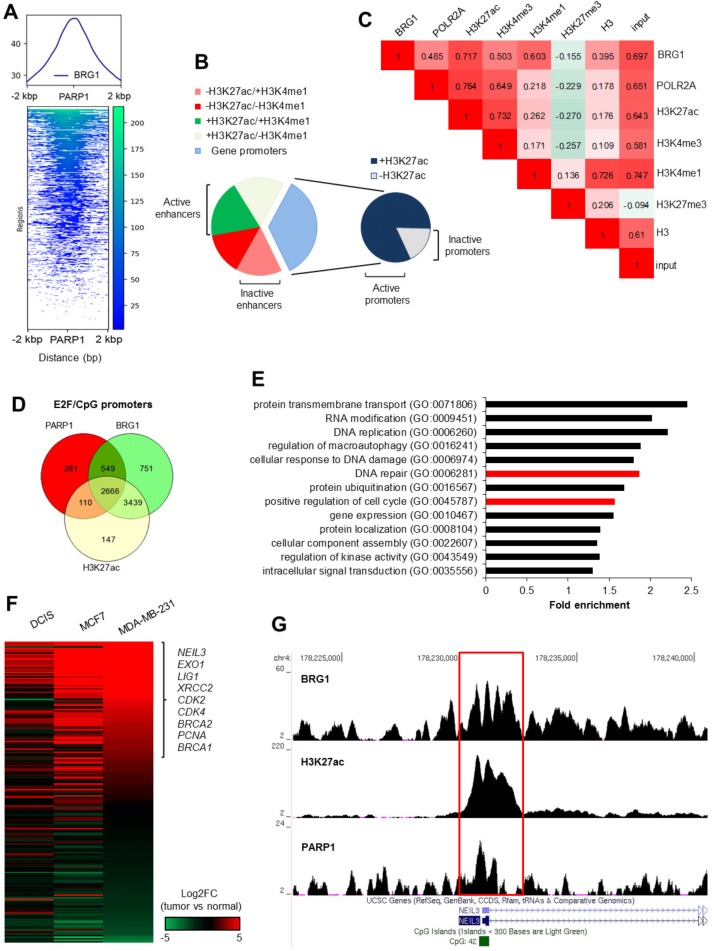
PARP1 co-occurs with BRG1 at the promoters of transcriptionally active genes in MDA-MB-231 cells. (**A**) An example computeMatrix/plotProfile plot of ChIP-Seq data for BRG1. BRG1 peaks are centered at PARP1-enriched regions in the genome of the MDA-MB-231 cell. (**B**) Association of PARP1/BRG1 peaks with gene regulatory regions shows a prevailing occurrence at the active gene promoters. Enhancers emerged based on H3K27ac/H3K4me1 status, whereas promoters were assumed as TSS ± 2 kbp. PARP1/BRG1-enriched regions were assigned to particular groups and quantified by bedtools Intersect intervals. (**C**) BRG1 distribution at the PARP1 peaks reveals a relatively strong correlation with histone markers and with POLR2A, which is typical for actively transcribed genes (multiBamSummary/plotCorrelation; PARP1 peaks in bed for scoring). (**D**) Venn diagram showing that a high proportion of PARP1 positive gene promoters are characterized by the presence of BRG1 and strong acetylation of H3K27. (**E**) Triple-positive gene promoters identified in (**D**) represent a functional association with numerous intracellular processes. GO-enriched terms were identified in AmiGO2. (**F**) Differential expression of genes from two selected GOs, i.e., DNA repair and positive regulation of the cell cycle (marked in red in (**E**)) in cancer cells (DCIS – ductal carcinoma in situ, pre-invasive malignancy of the breast and two breast cancer cell lines: MCF7 and MDA-MB-231) versus normal cells, as quantified by Cuffdiff. The heatmap shows Log2 of the calculated fold change (Log2FC; cancer versus normal cells). (**G**) UCSC Genome browser visualization of PARP1, BRG1, and H3K27ac enrichment using Model-based Analysis of ChIP-Seq data (MACS) (bigwig) at the NEIL3 promoter.

BRG1 was distributed and perfectly centered on genomic regions enriched in PARP1 (Figure 2A). BRG1/PARP1 peaks appeared predominantly at the gene regulatory regions, i.e., at the promoters and enhancers (Figure 2B; definitions of terms are given in Section 4.9), where BRG1 showed a relatively strong correlation with histone modifications, which marked open chromatin and transcriptionally active regions (Figure 2C). Focusing only at the E2F/CpG-driven gene promoters (Table S2), most of the PARP1 peaks (80.2%) were localized at the BRG1/H3K27ac-featured regions adjacent (±2 kbp) to the transcription start site (Figure 2D). This agrees with our and others’ reports, where BRG1 has been documented to be associated with promoters of actively transcribed genes, characterized by histone marks, which are permissive for transcription, and the presence of CpG and E2F binding motifs. Our previous findings ascribed BRG1 as a master regulator of some H3K27ac positive promoters, which is essential for the initiation of mRNA synthesis [11,12].

PARP1/BRG1/H3K27ac-positive promoters represent genes that are functionally assigned to numerous processes that are crucial for the maintenance of intracellular homeostasis (Figure 2E; Table S3). To further investigate the molecular mechanisms that underlie the possible functional cross-talk between PARP1 and the BRG1-based SWI/SNF complex, we chose genes attributed to two groups, namely, DNA repair and positive regulation of the cell cycle. These groups were chosen because we recently selected them for an in-depth examination and explained a mutual interdependence between gene expression, BRG1 activity, promoter features, and cell proliferation status [12]. Most genes in the considered groups were over-expressed in at least one of the two fast-proliferating breast cancer cell lines when compared to normal cells derived from primary breast tissue (Figure 2F and Table S4). Particular attention was paid to three of our previously studied genes, *cyclin dependent kinase 4* (*CDK4*), *DNA ligase 1* (*LIG1*), and *nei like DNA glycosylase 3* (*NEIL3*). The transcription of these genes was driven by E2F/CpG/H3K27ac-positive promoters, which were found to be enriched in PARP1 in addition to BRG1 (Figure 2G). The molecular mechanism driving the transcription in MCF7 and MDA-MB-231 cells involved cooperation between EP300, which acetylated histones at the studied gene promoters, and BRG1, which evicted marked nucleosomes in proliferating cells [11,12].

### 2.3. PARP1 Conditions Transcriptionally Permissive Chromatin Structure at BRG1/EP300-Dependent Genes

**Figure 3:**
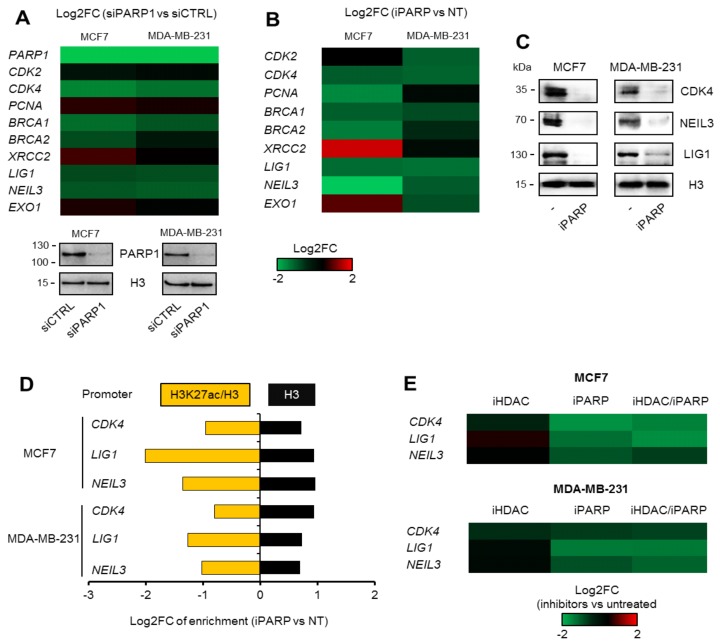
ADP-ribosylation confers open chromatin structure at the gene promoters. (**A**) PARP1 silencing leads to the suppression of most genes in MCF7 and MDA-MB-231 cells that feature PARP1/BRG1/H3K27ac-positive promoters. mRNA was compared 48 h after cell transfection with siCTRL and siPARP1. Log2 of the calculated fold change (Log2FC) shows gene expression in cells treated with inhibitors and normalized to untreated cells. The silencing of PARP1 was confirmed by Western blotting (below heatmap), and H3 was used as a loading control. A similar effect was observed upon PARP1 inhibition with olaparib (iPARP; 48 h) at both the mRNA (**B**) and protein level. (**C**) Representative pictures of protein detection by Western blotting. (**D**) Analysis of structure of selected PARP1-dependent gene promoters revealed a considerable loss of histone acetylation, but increased nucleosome density upon PARP1 inhibition for 24 h. Quantification was carried out by ChIP-qPCR, and data for specific antibodies were normalized first to 10% of the corresponding input and then to untreated control cells. (**E**) The iPARP effect on gene transcription with HDAC activity deficiency (cells were treated with both inhibitors for 48 h) was studied by real-time PCR. Results are presented as Log2 of the calculated fold change (inhibitor versus untreated; Log2FC).

To verify the PARP1 contribution to the transcription of genes that are concomitantly controlled by BRG1–EP300 complexes (described by Sobczak et al. [12]), we targeted PARP1 with siRNA in both studied breast cancer cell lines and measured the mRNA levels of the selected genes representing two gene ontologies (Figure 3A). PARP1 silencing resulted in considerable suppression of most genes, with *XRCC2* being the only exception that responded to PARP1 deficiency, but not to inhibition with olaparib (iPARP, pan-PARP inhibitor) with increased transcription in MCF7 cells. Furthermore, to check whether the observed PARP1 impact on gene transcription required enzymatic activity, cells were treated with olaparib, a PARP inhibitor. Loss of this enzymatic activity phenocopied PARP1 protein deficiency for *CDK4*, *BRCA1*, *LIG1*, and *NEIL3*, indicating that poly-ADP-ribosylation plays a key role in maintaining a high transcription rate of the considered genes (Figure 3A,B, Table S5). Enzyme inhibition led to a dramatic decline in the cellular abundance of CDK4, LIG1, and NEIL3 proteins (Figure 3C). PARP1 deficiency repressed the transcription of the three studied genes: *CDK4*, *LIG1,* and *NEIL3* comparably to iSWI/SNF and iEP300 (no synergistic effect was observed according to Supplementary Figure S1 and Table S5; only *LIG1* in MDA-MB-231 cells responded with enhanced gene repression after the treatment of siPARP1 transfected cells with iEP300 and iSWI/SNF), suggesting that this enzyme operates with the same, previously studied regulatory mechanism that utilizes the activity of BRG1 and EP300 at the three gene promoters considered [11,12], and that PARP1 may positively affect at least one of the chromatin-remodeling enzymes. This set of data suggests that PARP1 may also operate independently of EP300 and BRG1 (e.g., as a repressor of *XRCC2* in MCF7 cells).

Since EP300 and BRG1 drive gene transcription by respectively acetylating and displacing histones, to allow assembly of the transcriptional machinery, we focused on nucleosome acetylation status and density as possible readouts of PARP1 activity to identify the molecular basis of the observed effect of poly-ADP-ribosylation on BRG1–EP300-dependent gene expression. PARP inhibition with olaparib led to a substantial loss of histone acetylation and was associated with an increase in histone density (Figure 3D; H3 enrichment and status of H3K27ac for each of the studied promoters can be found in Table S5: sheet: LIG1, NEIL3, CDK4 ChIP)); the *XRCC1* promoter was used as a negative control since it lacks PARP1 (Figure S2; Table S5: sheet: XRCC1 ChIP). This finding confirmed that ADP-ribosylation impacts BRG1–EP300 complexes in rapidly proliferating cells and defines the output of the considered chromatin-remodeling functional unit. 

Knowing that BRG1 and EP300 co-occur at the studied gene promoters with HDAC1, the observed PARP1 effect on histone acetylation and gene transcription may result from PARP1 interaction with either of the two enzymes, since the subtle balance between acetylase and deacetylase activity determines the BRG1-dependent chromatin structure [11,12]. Thus, we tested whether poly-ADP-ribosylation inhibited HDAC1 activity at the gene promoters by comparing gene expression in the presence of HDAC and PARP inhibitors (Figure 3E). First, HDAC1 did not reduce the transcription of any of the three genes, and second, cell treatment with a mixture of both inhibitors suppressed *CDK4*, *LIG1*, and NEIL3 in a similar way to iPARP only (PARP and HDAC activities had no synergistic impact on the gene expression; Table S5). This indicates that olaparib does not inhibit HDAC1 (or any other histone deacetylase, since we used a pan-HDAC inhibitor) activity from poly-ADP-ribosylation-dependent inhibition at the studied gene promoters.

### 2.4. Poly-ADP-Ribosylation of EP300 Drives BRG1–EP300-Dependent Gene Transcription

Identified PARP1 interaction with BRG1 frequently occurred at highly acetylated gene promoters (Figure 2B). The fact that at least some of them were previously confirmed to be enriched in BRG1–EP300 functional complexes [12] prompted us to check if EP300 interacted with PARP1 and if any of BRG1–EP300 components could undergo ADP-ribosylation in proliferating breast cancer cells.

**Figure 4:**
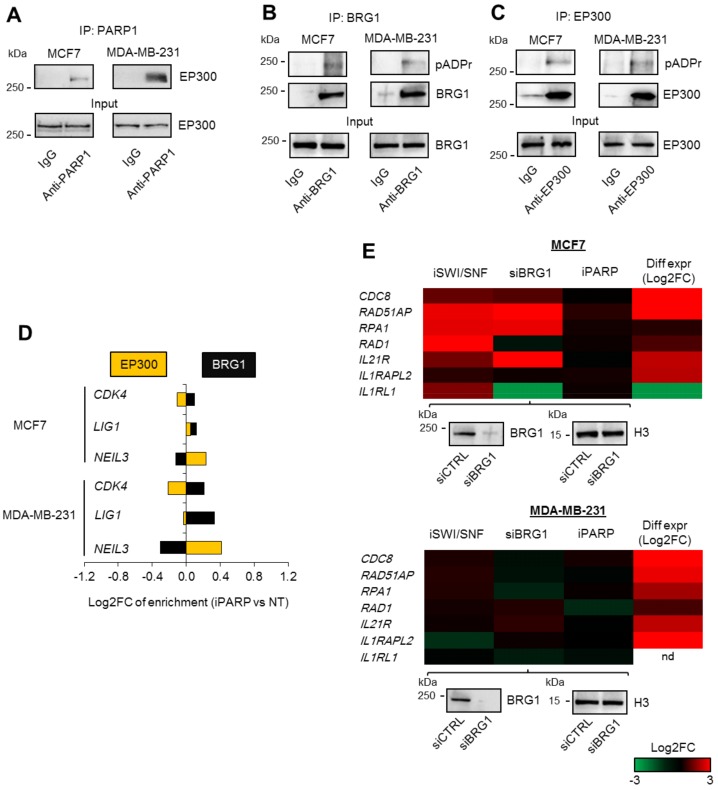
ADP-ribosylation of EP300 drives BRG1-dependent gene transcription. (**A**) The PARP1 interaction with EP300 was confirmed by visualization of EP300 by Western blot in PARP1 immunoprecipitates. (**B**) ADP-ribosylation of BRG1 was detected in BRG1 pull-downs by Western blot. (**C**) ADP-ribosylation of EP300 was detected as in (**B**) in EP300 pull-downs. (**D**) The effect of ADP-ribosylation on EP300 and BRG1 association with gene promoters in breast cancer cells was studied by ChIP-qPCR. Cells were supplemented with iPARP for 24 h prior to cell fixation. Data for anti-EP300 and anti-BRG1 were normalized to 10% input and then to untreated cells (Log2 enrichment vs. control cells). (**E**) The lack of contribution of ADP-ribosylation to the transcription of genes characterized by BRG1/PARP1 (no EP300) positive promoters was verified by comparing mRNA (real-time PCR) between the studied groups. For inhibitors, Log2FC was normalized to untreated cells, while data for BRG1 silencing were normalized to cells transfected with control siRNA. The efficiency of BRG1 silencing is confirmed by Western blot (below heatmaps). The column with differential expression shows gene transcription in MCF7 and MDA-MB-231 cell lines normalized to normal cells (data from RNA-Seq).

Analysis of PARP1 pull-downs by Western blotting confirmed the physical interaction between PARP1 and EP300 (Figure 4A). Poly-ADP-ribose chains were detected in both immunoprecipitated enzymes, i.e., BRG1 and histone acetyltransferase, in the studied cancer lines (Figure 4B,C), thereby providing further evidence for a possible PARP1 role in the regulation of transcriptional activity of BRG1–EP300 complexes. Bearing in mind that the dependence of gene transcription on BRG1 and EP300 is conditioned by the association of enzymes with their gene promoters and then by the catalytic activity of chromatin-bound enzymes, we tested whether poly-ADP-ribosylation affected BRG1 and EP300 levels at the investigated promoters (Figure 4D). None of the studied genomic regions responded to PARP1 inhibition with substantial displacement or recruitment of chromatin remodeling enzymes, suggesting that poly-ADP-ribosylation determines the activity of enzymes rather than their occurrence at the gene promoters (Figure 4D, Table S5). The poly-ADP-ribosylation of EP300 enabled acetyltransferase activity that led to intensive nucleosome acetylation and eviction by BRG1, since cell treatment with a PARP inhibitor resulted in a dramatic loss of EP300-dependent acetylation of the studied gene promoters (Figure 3D, Table S5), without an apparent effect on the association of EP300 with the gene promoters and the HDAC1 role in gene transcription (Figure 4D and Figure 3E).

To check if poly-ADP-ribosylation of BRG1 directly conditioned BRG1 activity and BRG1-dependent transcription, we tested the impact of the PARP1 inhibitor on the transcription of genes that are over-expressed in cancer cells and characterized by the occurrence of PARP1 and BRG1 at their promoters, but without considerable nucleosome acetylation (Figure 4D and Table S2). *IL1RL1* served as an example of repressed genes in MCF7 cells. Surprisingly, all of the genes found to be over-expressed that were considered in this experiment responded to SWI/SNF inhibition and silencing with increased transcription (Figure 4E, Table S5), suggesting that EP300 co-distribution with BRG1 might be a hallmark of gene promoters characterized by the pro-transcriptional activity of BRG1-based SWI/SNF complexes. However, this hypothesis requires further examination of a wider range of genes, especially because the considerable inhibitory role of BRG1 on gene transcription was observed in only one cell line. This finding also stresses the differences in gene transcription control in the two chosen cell lines. In any case, PARP1 was not involved in the transcriptional regulation of genes suppressed by the SWI/SNF complex; only one repressive effect was found for *RAD1* in MDA-MB-231 cells. Together, these data indicate that PARP1 co-regulates activity of promoter-bound BRG1–EP300 complexes, and that poly-ADP-ribosylation of EP300 is required to enable the BRG1-dependent eviction of acetylated nucleosome, and therefore the transcription of genes involved in key intracellular processes, such as cell division and the removal of DNA damage in breast cancer cells. This molecular mechanism of PARP1 action in BRG1–EP300 complexes is further supported by our previous findings, in which BRG1 emerged as a reader of nucleosome acetylation [11,12]. Thus, a low histone acetylation caused by PARP inhibition prevents histone eviction by BRG1.

## 3. Discussion

Malignant transformation directs cellular changes and adaptive responses toward new requirements that cancer cells face during growth and metastasis. As long as the activation of oncogenes, loss of cell cycle checkpoint control, and impaired DNA repair capacity favor carcinogenesis, genome integrity and cell cycle re-entrance will eventually become threatened due to increased energy demand, mild but persistent oxidative stress, and the modulation of signaling cascades necessary for tumor growth and the invasion of other tissues [17,18]. The altered expression of many gene products in response to cell transformation is affected by reprogramming the epigenome, resulting in changes in the transcription and reconstruction of the cellular proteome to meet emerging needs [19]. According to our findings, the activation of *PARP1* transcription as a consequence of the transition of a cell from quiescence to proliferation may help the cancer cell gain the necessary adaptive physiology by acting at the genomic level in two ways: directly, by contributing to DNA repair machineries, and indirectly, by affecting the transcription of BRG1–EP300 targets, among others, which enable the cancer cell to rapidly divide and resist DNA damaging agents [12,20,21,22]. Although the first aspects of this mechanism have been relatively deeply explored and the regulatory roles of PARP1 regarding the removal of DNA lesions and the transition between consecutive cell cycle phases in various modes—for example, the modulation of SP1 activity, H1 displacement at proliferation-relevant genes, and nuclear retention of PKM2—have been documented, the detailed molecular mechanism in regard to functionally linked genes has not been identified until now [23,24,25]. In this study, PARP1 was shown to be an active component of the transcription machinery that drives BRG1-EP300-dependent gene expression by the poly-ADP-ribosylation of EP300 in breast cancer cells. PARP1 was highly enriched at gene promoters characterized by the occurrence of not only BRG1 and EP300, but also E2F binding motif(s) and CpG islands. Since these features apply to many functionally linked genes, the role of PARP1 in defining the breast cancer phenotype at the transcription level is likely to go far beyond cell cycle progression and the removal of DNA damage. As shown in Figure 2E, triple-positive PARP1/BRG1/H3K27ac promoters represent the genes responsible for signal transduction and autophagy. Once poly-ADP-ribosylation is proved to be a co-activator of these genes, PARP inhibitors may be important to consider for pharmacological interventions that target and suppress mediators of pro-survival cascades at the genomic level. The role of poly-ADP-ribosylation in the fine-tuning of numerous intracellular processes simultaneously allows the maximization of the effectiveness of PARP1 inhibitors in rendering cancer cells vulnerable to anticancer drugs, which challenge PARP1-dependent or concurrent intracellular routes. Bearing in mind that the BRG1/EP300 complex was shown to operate at gene promoters in proliferating breast cancer cells and human macrophages due to an association with E2F transcription factors [12,26], the same PARP1-dependent mechanism of transcription control likely applies to other tumors, since the cell cycle status conditions both PARP1 expression and the activity of BRG1–EP300 complexes. Similarly, the energy status of proliferating cells demands a high ATP concentration; thus, the NAD^+^/NADH redox ratio determines, for example, that PARP1 activity is five times higher in cancer cells than in normal, non-transformed cells [27]. Therefore, it might be possible to adapt PARP1 inhibitors for the modulation of intracellular processes in a wider range of cancers.

This study revealed a new mechanism that defines cancer cell responses to DNA lesions in a poly-ADP-ribosylation-dependent manner. Although the direct contribution of PARP1 to pathway repair at sites of damage is well acknowledged [28,29], we showed for the first time an impact on the repair mechanisms that is distant, gene promoter-related, and independent of lesion location. In this context, PARP inhibitors could be used to suppress the transcription of genes characterized by promoters enriched in PARP1, BRG1, and EP300, which represent executors that are crucial for BER, SSBR, NER, MMR, HR, and alt-NHEJ, since products of only the considered genes, i.e., *BRCA1/2*, *LIG1,* and *NEIL3,* contribute to more than one repair pathway [30,31]. Another benefit of using PARP inhibitors as DNA repair modulators acting at the level of the epigenome comes from the observation that poly-ADP-ribosylation is a co-activator of cell cycle-dependent genes that are simultaneously controlled by BRG1 and EP300, and are mostly over-expressed in the studied breast cancer cells, thus providing some selectivity toward this group. PARP1 was also found at gene promoters occupied by BRG1 alone, but cell treatment with olaparib did not reveal a considerable impact on the expression of these genes. Notably, the PARP1/BRG1 promoter response to an SWI/SNF inhibitor that is capable of impeding the activity of both ATPases (Figure 4D) suggested that (a) EP300 has a co-activating role with BRG1 at the BRG1/EP300 promoters, and/or (b) the role of particular ATPases may be determined by EP300, and the lack of acetyltransferase may switch BRG1 to BRM activity. Although the scope of iSWI/SNFs is broader than that of iPARPs, the unexpected reaction of the chromatin to the loss of BRG1 and BRM activity may result in a coinciding repression and up-regulation of genes assigned to one regulatory circuit. Discrete inhibitors of the two ATPases mentioned, as well as further study on the mutual interdependence between these two enzymes, are needed for future clinical applications. Similarly, inhibitors of EP300 modulate the transcription of a significant number of genes at different levels; at the genome level by preventing acetylation of transcription co-factors and nucleosomes, and at the signaling cascade level by modifying signal transducers [32,33]. This all results in even less specificity toward the desired gene pool, and underlines the importance of the possible applications of PARP1 inhibitors. Some PARP-1 and PARP-2 inhibitors, such as olaparib, niraparib, rucaparib, veliparib, and talazoparib, which are small-molecule NAD^+^ mimetics, are currently being studied in later-stage clinical trials or are already approved for breast and ovarian cancer treatment with deleterious germline *BRCA1* and *BRCA2* mutations, which predispose women to develop triple-negative and hormone-receptor-positive, human epidermal growth factor receptor 2-negative breast cancers, respectively [34,35]. Since PARP inhibitor monotherapy strategies are effective in cancers with homologous recombination repair defects and are relatively well-tolerated by patients, they can be considered for the treatment of a wider range of cancers, both in combined therapies, due to the well-established fact that these drugs sensitize cells to DNA-damaging chemotherapy and radiation therapy, or as an alternative to taxanes and a supplement to anthracyclines [36,37,38]. However, numerous phase I clinical trials utilizing a combination of cytotoxic chemotherapy with PARP inhibitors failed to confirm any beneficial effects of such combinations. Therefore, the use of these drugs in adjuvant or neoadjuvant settings may need substantial revision, while also taking into consideration the myelosuppressive effects of PARP inhibitors and the careful selection of anticancer agents in combination with DNA repair inhibitor(s). Nevertheless, the long list of promoters of functionally related genes that are enriched in PARP1 presented in this manuscript suggest the likely involvement of this enzyme regarding the modulation of other intracellular processes at the transcription level. These findings open the gate for new ideas and concepts regarding anticancer approaches, which require verification first in cell and animal models.

The described contribution of PARP1 to the regulation of BRG1–EP300 activity emphasizes the role of PARP1 in chromatin remodeling. Although a number of papers have documented this enzyme as a direct or indirect regulator of chromatin structure in a context-dependent fashion, none have provided an overall mechanism for functionally linked gene sets in particular. Transcription in cells is shaped by the poly-ADP-ribosylation of nucleosomes, histone writers and erasers (KDM5B), transcription factors (e.g., C/EBPβ), or POL2 regulating co-factors (NELF), as well as physical, activity-independent interactions with gene promoters that define chromatin composition (LSD1, EP300) [13,14,15,16,39,40]. However, DNA motifs or chromatin signatures, which determine PARP1 distribution in the genome, remain unidentified. According to Gibbson, PARP1 and ADP-ribosylation correlated with histone markers (H3K4me3 and H3K27ac) featuring actively transcribed genes and with POL2 pausing machinery in embryonic fibroblasts of mice [40]. These findings agree with our own, in which PARP1 was enriched at highly acetylated CpG islands, allowing immediate POL2 pausing or release in response to received signals [11,12]. The association of PARP1 with GC-rich regions impedes the identification of the PARP1-specific motif in promoter sequences. Since only 19% of PARP1 peaks in the genome of MDA-MB-231 cells occurred at BRG1 and H3K27ac negative promoters, and less than 3% outside of BRG1 peaks, these two features of promoters, together with E2F-binding motifs and CpG islands, seem to direct the enzyme to its destination regarding chromatin, whereby the poly-ADP-ribosylation of BRG1 and EP300 enables gene expression.

## 4. Materials and Methods

### 4.1. Materials

Two epithelial, breast cancer cell lines, derived from metastatic sites, MCF7 (estrogen and progesterone receptors-positive, HER2-negative) and MDA-MB-231 (triple negative) were purchased from ATCC and Sigma Aldrich (Poznan, Poland), respectively. DMEM high glucose with L-glutamine with sodium pyruvate for MCF7, fetal bovine serum, and antibiotics (penicillin and streptomycin) were from Biowest (CytoGen, Zgierz, Poland), L15 Medium for MDA-MB-231, iSWI/SNF (PFI-3), iHDAC (sodium butyrate), anti-rabbit IgG (A0545) and anti-mouse IgG (A4416) (whole molecule)–peroxidase antibody produced in goat, BLUeye prestained protein ladder (#94964), oligonucleotides for real-time PCR, SIGMA*FAST*™ Protease Inhibitor Tablets (PIC) were from Sigma Aldrich (Poznan, Poland). iPARP (olaparib, AZD-2281) was from Cayman Chemical (Biokom, Janki/Warsaw, Poland). Lipofectamine RNAiMAX, OptiMem, Dynabeads™ Protein G, glycogen, High-Capacity cDNA Reverse Transcription Kit, SuperSignal™ West Pico Chemiluminescent Substrate, TRI Reagent™, and RNase A were from Thermofisher Scientific (Thermofisher Scientific, Warsaw, Poland). KAPA HiFi™ HotStart ReadyMix (2×) from KapaBiosystems and Takyon™No ROX SYBR Core Kit blue dTTP from Eurogentec were purchased from Polgen (Lodz, Poland). EvaGreen^®^ Dye, 20X in water was purchased from Biotium (Corporate Place Hayward, Fremont, CA, USA). WB antibodies: anti-DNA Ligase I (sc-271678), anti-CDK4 (sc-23896), anti-NEIL3 (sc-393703), anti-pADPr (sc-56198), siPARP1 (sc-29437), and gallotannin were purchased from Santa Cruz Biotechnology (AMX, Lodz, Poland). ChIP grade antibodies: normal rabbit IgG (#2729), anti-ARID1A (#12354), anti-SMARCC2 (#12760), anti-BRG1 (#49360), anti-H3K27ac (#4353), anti-histone H3 (#4620), and anti-PARP1 (#9532) were purchased from Cell Signaling Technology (LabJOT, Warsaw, Poland). Human Cytokine and Chemokine Receptor Primer Library (HCCR-I) were from RealTime Primers (Prospecta, Warsaw, Poland). For the mass spectrometric analysis, all materials were from Thermo Fisher Scientific (Loughborough, UK) unless otherwise indicated. Porcine Trypsin (Trypsin Gold Mass Spectrometry Grade) was from Promega (Southampton, UK), and general use Protease Inhibitor Cocktail (P2174) was from Sigma-Aldrich (Poole, UK).

### 4.2. Cell Culture and Treatment with Inhibitors

MCF7 were cultured in DMEM supplemented with 10% FBS, penicillin/streptomycin (50 U/mL and 50 µg/mL, respectively) in 5% CO_2_, whereas MDA-MB-231 was cultured in F15 medium supplemented with 15% FBS and penicillin/streptomycin (50 U/mL and 50 µg/mL, respectively) without CO_2_ equilibration. After freezing and thawing, cells were cultured in DMEM as described for MCF7 cells. iSWI/SNF (10 µM; PFI-3), iPARP1 (olaparib, 1 µM), and iHDAC1 (sodium butyrate, 250 µM) were added to cells 48 h prior to analysis.

### 4.3. Quantification of Gene Expression

mRNA quantification was conducted as described in Pietrzak et al. [11] using Takyon™ No Rox SYBR^®^ MasterMix dTTP blue (Eurogentec—from local distributor—Polgen, Lodz, Poland) and CFX96 C1000 Touch (BioRad, Warsaw, Poland) for real-time PCR. The median average of ACTB, GAPDH, and B2M were used for normalization. Data in figures are shown as Log2FC with respect to untreated control or to siCTRL (indicated in figures or figure legends).

For protein detection, cell lysates were processed as previously described and visualized using SuperSignal™ West Pico Chemiluminescent Substrate. Pictures were acquired with ChemiDoc-IT2 (UVP, Meranco, Poznan, Poland).

The following primer sets were used for the quantification of gene expression: *CDK2*, 5′-CAGGATGTGACCAAGCCAGT-3′ (forward) and 5′-TGAGTCCAAATAGCCCAAGG-3′ (reverse); *CDK4*, 5′-CTGGTGTTTGAGCATGTAGACC-3′ (forward) and 5′-AAACTGGCGCATCAGATCCTT-3′ (reverse), *XRCC2*, 5′-TCGCCTGGTTCTTTTTGCA-3′ (forward) and 5′-TCTGATGAGCTCGAGGCTTTC-3′ (reverse), *BRCA2*, 5′-CTTGCCCCTTTCGTCTATTTG-3′ (forward) and 5′-TACGGCCCTGAAGTACAGTCT-3′ (reverse), *LIG1*, 5′-CAGAGGGCGAGTTTGTCTTC-3′ (forward) and 5′-AGCCAGTTGTGCGATCTCTT-3′ (reverse), *EXO1*, 5’-AAACCTGAATGTGGCCGTGT-3′ (forward) and 5′CCTCATTCCCAAACAGGGACT-3′ (reverse), *NEIL3*, 5′-GGTCTCCACCCAGCTGTTAAAG-3′ (forward) and 5′-CACGTATCATTTTCATGAGGTGATG-3’ (reverse), *PCNA*, 5’-TCTGAGGGCTTCGACACCTA-3’ (forward) and 5′- TTCTCCTGGTTTGGTGCTTCA-3′ (reverse); *BRG1*, 5′-AAGAAGACTGAGCCCCGACATTC-3′ (forward) and 5′-CCGTTACTGCTAAGGCCTATGC-3′ (reverse), *BRCA1*, 5′-TGCCCACAGATCAACTGGAA-3′ (forward) and 5′- CACAGGTGCCTCACACATCT-3′ (reverse); *ACTB*, 5′- TGGCACCCAGCACAATGAA-3′ (forward) and 5′-CTAAGTCATAGTCCGCCTAGAAGCA-3′ (reverse); PARP1, 5′- AAGCCCTAAAGGCTCAGAACG-3′ and 5′-ACCATGCCATCAGCTACTCGGT-3′. *GAPDH* and *B2M* were from the Human Toll-like Receptor Signaling Primer Library (HTLR-I, RealTime Primers – from local distributor - Prospecta, Warsaw, Poland)).

### 4.4. PARP1 Co-Immunoprecipitation for Mass Spectrometry

2 × 10^7^ MCF7 cells were trypsinized, washed 3× with cold PBS, and lysed on ice in hypotonic buffer (50 mM HEPES-KOH, 10 mM NaCl, 1 mM EDTA, 10% glycerol, and 0.5% NP-40, 0.25% TritonX-100). Nuclei were washed twice in PBS supplemented with protease inhibitor cocktail (10× stock added at 10% of volume, Sigma P2714 General Purpose) and fixed in 1% formaldehyde in PBS on stirrer for 10 min at room temperature. Formaldehyde was quenched by the addition of 125 mM oglycine, and after 20 min of incubation, nuclei were washed twice in 10 mM Tri-HCl (pH = 8.0), 100 mM NaCl, and 1 mM EDTA, and finally resuspended in 10 mM Tri-HCl (pH = 8.0), 150 mM NaCl, 1 mM EDTA, 0.05 Na-deoxycholate, and 0.25% N-laurosarcosine. Nuclei were sonicated until the solution was transparent, TritonX-100 was added to 1% and then centrifuged (10,000× g, 4 °C, 10 min) to remove insoluble material. The supernatant was split into two samples, to which were added either control IgG–Dynabead or anti-PARP1–Dynabead conjugates (prepared by the incubation of 5 µg of antibody and 10 µL of magnetic beads in 10 mM Tri-HCl pH 8.0, 150 mM NaCl, 1 mM EDTA, 0.05 Na-deoxycholate, and 0.25% N-laurosarcosine for at least 15 min). After overnight incubation on a roller shaker at 4 °C, the supernatant was removed, and the beads were washed 5× with 50 mM HEPES-KOH (pH = 7.5), 500 mM LiCl, 1 mM EDTA, 1% NP-40, and 0.7% Na-deoxycholate; twice with 50 mM NaCl in TE buffer (10 mM Tris pH = 8.0, 1 mM EDTA); and twice with 50 mM ammonium bicarbonate using a magnetic stand. Then, the beads were incubated with trypsin (Promega Gold, 1 µg/µL) in 50 mM ammonium bicarbonate for 6 h at 36 °C, after which the supernatant was transferred to new tubes and dried in a SpeedVac vacuum concentrator (Eppendorf, Stevenage, UK).

### 4.5. Mass Spectrometry Analysis of PARP1 Co-Immunoprecipitates

Tryptic digest samples were resuspended in 25 µL of 2% acetonitrile in water and 0.5% formic acid. Peptides were separated and analyzed using a U3000 nanoflow LC system (Thermo) connected to a 5600 Triple ToF mass spectrometer (Sciex, Warrington, UK). Then, 10 µL of sample was loaded onto a 0.5 × 5 mm PepMap C18 trap, washed with buffer A (2% acetonitrile 98% water containing 0.5% formic acid) for 4 min, and then separated by a 90-minute gradient from 2% to 40% buffer B (98% acetonitrile, 2% water containing 0.5% formic acid) on a 0.075 × 150 mm PepMap C18 column (Thermo Fisher Scientific, Loughborough, UK)). MSMS data were collected for precursors of 2^+^ to 5^+^ charge state in the range m/z 350–1250 Th using a top 10 data dependent acquisition method, collecting MS data for 200 ms and MSMS data for 100 ms, with dynamic exclusion for 15 s and a standard rolling collision energy settings. MSMS data was collected in the range of m/z 50–2000 Th. All other settings were optimized for peptides using a standard mixture. Samples were run as biological replicates. MSMS data was analyzed using Progenesis QIP (Waters, Manchester, UK) for label-free quantitative analysis and Mascot (Matrix Science, London) for protein identification. Data was loaded as .wiff files into Progenesis QIP, automatically aligned, and peak picked using the default settings; then, the alignment was manually improved where necessary. Default settings were used for peak picking, and Hi-N was used for quantification. Data was exported to Mascot using the default settings. For the Mascot analysis, the SwissProt Mammalian database (2018_09) was searched, allowing 50 ppm error for MS and 100 ppm for MSMS data, two missed cleaves, methionine oxidation as a variable modification, and an overall FDR of <1%. Data was re-imported into Progenesis QIP for further quantitative analysis. Protein identifications were deemed significant if more than two peptides were identified with an overall confidence score greater than 50, but more stringent criteria were applied for proteins to be further investigated. Quantification data was considered significant where the ANOVA *p*-value was less than 0.05, the fold change was greater than 2, and the highest mean was in the PARP immunoprecipitation.

### 4.6. Co-Immunoprecipitation and Western Blot

MCF7 and MDA-MB-2331 cells were lysed in 20 mM Tris-HCl (pH = 7.5), 75 mM KCl, 5 mM MgCl_2_, 0.2 mM EDTA, 10% glycerol, 0.1% Tween20, and PIC (IP buffer); sonicated with the ultrasonic homogenizer Bandelin Sonopuls (HD 2070; 10 impulses, 60%); and centrifuged (10,000× *g*, 4 °C, 10 min). Supernatant was incubated with anti-PARP1, anti-BRG1, anty-EP300, and corresponding IgG at 4 °C for 2 h. For another 1 h, lysates were added with Dynabeads (5 µL); then, they were washed 5× with the IP buffer and once in 50 mM NaCl in TE buffer (10 mM Tris pH = 8.0, 1 mM EDTA). Beads were suspended in gel loading buffer supplemented with 5% β-mercaptoethanol, and heated at 70 °C for 10 min. Beads were collected on a magnetic stand and supernatant was separated by PAGE. BRG1, ARID1A, SMARCC1, SMARCC2, PARP1, EP300, H3, and poly-ADP-ribose were detected on nitrocellulose membranes after overnight staining with corresponding antibodies. For the detection of poly-ADP-ribosylation, cells were lysed and processed in the presence of a PARG inhibitor: 0.5 mM gallotannin.

Each immunoprecipitation followed by Western blot was repeated in three biological replicates. Each time, the striking difference in protein level being detected was observed between IgG (weak or lack of signal) versus anti-PARP1 (or anti-BRG1; strong and clear bands). Representative images were taken for figures.

### 4.7. Chromatin Immunoprecipitation 

The immunoprecipitation of chromatin-bound proteins and histones was carried out according to the protocol previously described [11]. For the quantification of H3K27 acetylation, cells were lysed and processed in the presence of iHDAC (0.5 mM). Fragments spanning PARP1/BRG1/H3K27ac sites in selected gene promoters were amplified using KAPA HiFi™ HotStart ReadyMix supplemented with EvaGreen^®^ Dye and 4% DMSO. Primers for *CDK4*, *LIG1, NEIL3*, and *XRCC1* promoters were as follows: *CDK4* prom, 5′-ATAACCAGCTCGCGAAACGA-3′ and 5′-AGAGCAATGTCAAGCGGTCA-3′, *LIG1* prom, 5′-AACACACTCAGATCCGCCAG-3′ and 5′-GCTTCCACCGATTCCTCCTC-3′, *NEIL3* prom, 5′-GTAGGGAGCGACCTCAACAG-3′ and 5′-AGTACAGCCTGGTCCTTCCA-3′, *XRCC1* prom, 5′- TGGCCAGAAGGATGAGGTAG-3′ (forward) and 5′-AGGAAACGCTCGTTGCTAAG-3′ (reverse).

### 4.8. Transient Gene Silencing

For PARP1 and BRG1 silencing, MCF7 and MCD-MB-23 were seeded at the density of 100,000 cells per well, transfected on the following day with RNAiMAX-siRNA complexes (3 µL of transfection reagent and 20 nmol siRNA incubated in OptiMem for 20 min). The silencing was confirmed by real-time PCR and Western blot 48 h after cell transfection.

### 4.9. ChIP-Seq Analysis in Galaxy Version 19.05.dev [41]

The following, publically available, generated by other groups and deposited in the *PubMed* Central Database data from MDA-MB-231 cells were taken for ChIP-Seq analysis: PARP1—GSM1517306 (SRR1593959), BRG1—GSM1856026 (SRR2171350), GSM1856027 (SRR2171351), and GSM1856028 (SRR2171352), H3K27ac—GSM1855991 (SRR2171311) and GSM1855992 (SRR2171312); H3K4me3—GSM1700392 (SRR2044734), H3K4me1—GSM2036932 (SRR3096750 and SRR3096751), H3K27me3—GSM949581 (SRR513994), H3K9ac—GSM1619765 (SRR1820123 and SRR1820124), H3—GSM2531568 (SRR5332805), POLR2A—GSM2309434 (SRR4240635), and Input—GSM1964894 (SRR2976843). FASTQ quality formats were unified to Sanger formatted with a FASTQ Groomer [26]. Reads were aligned to Human Genome version 19 using a Map with Bowtie for Illumina, and unmapped reads were filtered out. ChIP-seq peaks were called in MACS with a *p*-value cutoff for peak detection set at 10^−3^. BRG1 co-occurrence at the PARP1 peaks was monitored by computeMatrix/plotProfile (PARP1 peaks in bed were used as regions to plot, while mapped BRG1 reads were used for scoring) [42]. The co-distribution of BRG1, POLR2A, and histone modifications at the PARP1-enriched regions was studied by MulitBamSummary/plotCorrelation (regions of the genome were limited to PARP1 peaks in bed, mapped reads for scoring) [42]. Regions simultaneously enriched in BRG1, PARP1, H3K4me1, and H3K27ac were identified by returning intersects of the peaks in bed using bedtools Intersect intervals [43]. Regions localized outside of gene promoters and double positive for H3K4me1 and H3K27ac were assigned as active enhancers high in H3K4me1 and low in H3K27ac as inactive enhancers, while gene promoters were recognized by returning intersects of BRG1/PARP1 peaks and genomic regions ±2000 bp centered on TSS (overlapping intervals of both datasets). Promoters defined by high H3K27ac and associated with the presence of gene transcripts (RNA-Seq results for MDA-MB-231) downstream of corresponding TSS were assumed as active, while the lack of promoter acetylation marked inactive gene promoters. Genomic intervals for E2F (overlaps of E2F1 and E2F4) and CpG islands were taken from the UCSC main tables wgEncodeRegTfbsClusteredV3 and cpgIslandExt, respectively. Intersects of TSS ±2 kbp and CpG or E2F intervals were compared using bedtools Intersect intervals. The characteristics of gene promoters (occurrence of particular proteins and CpGs, histone modifications, E2F binding sites) were studied using Venn diagrams, which were created in http://www.interactivenn.net/ from gene lists. The annotation of PARP1/BRG1/H3K27ac promoters to biological processes was carried out in AmiGO2 (test type—binominal, correction—FDR) [44]. PARP1, BRG1, and H3K27ac peaks were visualized in the UCSC Genome Browser.

The following, publically available datasets for various breast cells, which have been generated by other groups, were downloaded from the PubMed Central Database data and used for ChIP-Seq analysis: data from normal breast, ductal carcinoma in situ (DCIS; pre-invasive malignancy of the breast), MCF7 and MDA-MB-231 cells were taken for RNA-Seq analysis: normal breast—GSM1695870 (SRR2040339), GSM1695872 (SRR2040341), GSM1695873 (SRR2040342), GSM1695874 (SRR2040343), GSM1695877 (SRR2040346), and GSM1695878 (SRR2040347); DCIS—GSM1695891 (SRR2040360), GSM1695898 (SRR2040367), GSM1695899 (SRR2040368), GSM1695882 (SRR2040351), GSM1695890 (SRR2040359), and GSM1695894 (SRR2040363); MCF7—GSM2422725 (SRR5094305), GSM2422726 (SRR5094306), GSM2422727 (SRR5094307), GSM2422728 (SRR5094308), GSM2422729 (SRR5094309), and GSM2422730 (SRR5094310); MDA-MB-231—GSM2422731 (SRR5094311), GSM2422732 (SRR5094312), GSM2422733 (SRR5094313), GSM2422734 (SRR5094314), GSM2422735 (SRR5094315), and GSM2422736 (SRR5094316). All samples were processed as described in Sobczak et al. [12]. Differential gene expression in cancer versus normal breast cells was calculated with Cuffdiff and shown as a heatmap for two selected GOs (positive regulation of cell cycle and DNA repair) [45].

## 5. Conclusions

In conclusion, our study describes a new mechanism regarding the regulation of the transcription of functionally linked genes that are significant for cancer cell physiology. Poly-ADP-ribosylation emerges as a chromatin remodeler that is capable of defining the activity of BRG1–EP300 complexes at the promoters of genes encoding cell cycle and DNA repair-promoting proteins. Although the PARP1 inhibitor olaparib emerges as a promising tool to modulate PARP1/BRG1/EP300-dependent gene expression due to its safety and well-established in vivo effects in cancer treatment, the functional impact of DNA repair gene repression in anticancer therapies requires further investigation. In any case, our study provides a basis for the search for new combinations of iPARPs with other compounds to increase the beneficial effects of anticancer approaches.

## Supplementary Materials

The following are available online at https://www.mdpi.com/2072-6694/11/10/1539/s1, Table S1. Protein List identified in PARP1 immunoprecipitates by Mass Spectrometry. Table S2. PARP1, BRG1, and H3K27ac distribution at the CpG/E2F positive gene promoters. Table S3. Full list of gene enrichment analysis for PARP1/BRG1/H3K27ac/E2F/CpG positive promoters. Table S4. Differential expression of proliferation and DNA repair genes in DCIS, MCF7 and MDA-MB-231 cells versus normal breast tissue. Table S5. Statistical analysis of data. Figure S1. Effect of PARP1 silencing, SWI/SNF inhibition, and EP300 inhibition on gene expression. Figure S2. Chip-qPCR data for effect of iPARP on the *XRCC1* promoter.
