# Supplementary material for: PARP1 Co-Regulates EP300–BRG1-Dependent Transcription of Genes Involved in Breast Cancer Cell Proliferation and DNA Repair
Source: Cancers (Basel). 2019 Oct 11;11(10):1539. doi: 10.3390/cancers11101539 (PMC6826995; doi:10.3390/cancers11101539)
Supplement: Supplementary file 1 [file cancers-11-01539-s001.zip › cancers-586282-supplementary - publish/Supplementary Figures.pdf]

cancers

Article

PARP1 Co-Regulates EP300–BRG1-Dependent Transcription of Genes Involved in Breast Cancer Cell Proliferation and DNA Repair

Maciej Sobczak [1], Andrew Pitt [2], Corinne M. Spickett [2] and Agnieszka Robaszkiewicz [1,*]

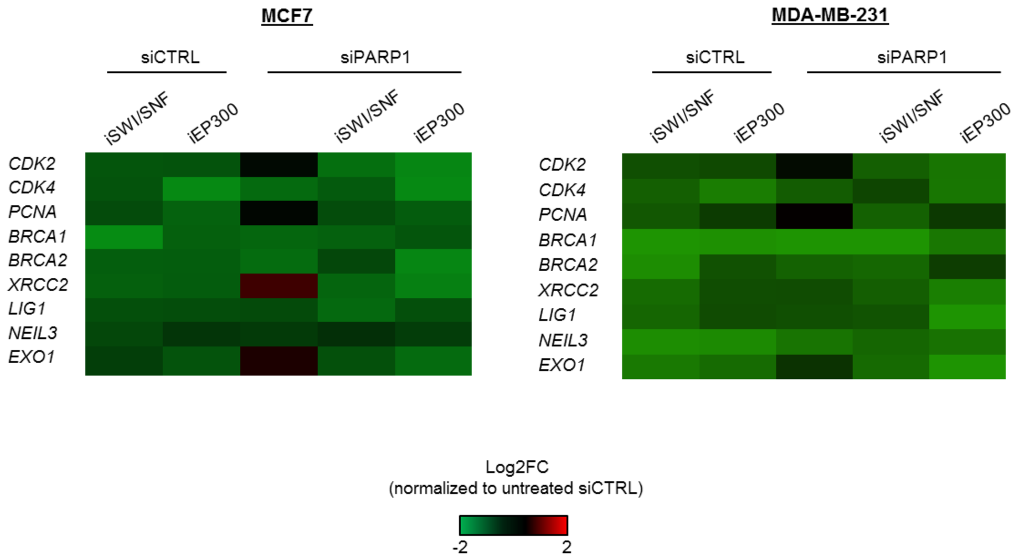

Figure S1. Effect of PARP1 silencing, SWI/SNF inhibition, and EP300 inhibition on gene expression. Real-time PCR was used to compare gene expression between the considered groups. Cell transfection with siRNA (24 h) was followed by treatment with inhibitors. After another 48 h, cells were collected and processed to isolate RNA.

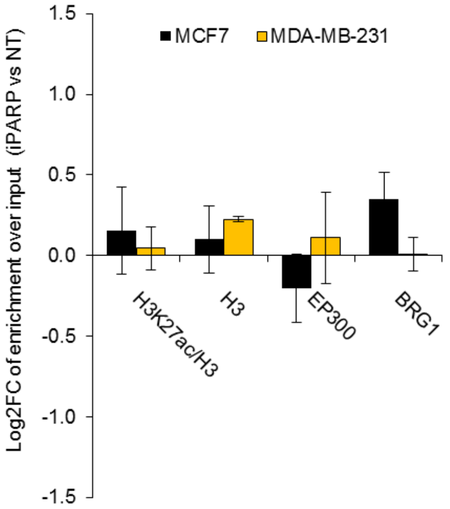

Figure 2. Chip-qPCR data for effect of iPARP on the *XRCC1* promoter. The highly acetylated promoter of *XRCC1*, that is also enriched in BRG1 (but not in PARP1), was chosen to check the possible effect of iPARP on chromatin structure. XRCC1 was highly expressed in MCF7 and MDA-MB-231 cells when compared to normal cells (Fig. 2F; Table S4). iPARP was added to cells 24 h prior to analysis.

Legends for Supplementary Tables

Table 1. Protein list identified in PARP1 immunoprecipitates by mass spectrometry.

Analysis of PARP1 co-immunoprecipitates by liquid chromatography mass spectrometry (LC-MS/MS), followed by quantification in Progenesis QIP, allowed the identification of a number of proteins interacting with PARP1 in MCF7 breast cancer cells, including the SWI/SNF complex component ARID1 (ARI1A), SMRC1, HDAC1, and SMCA4 (marked in red boxes). Anova and q values were generated by Progenesis QIP ($n = 6$ for PARP1 and $n = 4$ for the control). The confidence score is the Mascot-generated confidence score (MOWSE score) for the protein, and the peptide count is the number of peptides identified for the protein above the Mascot statistical cutoff. Infinity in max fold change means that the signal for the considered protein was not detected in the control immunoprecipitates (IgG).

Table 2. PARP1, BRG1, and H3K27ac distribution at the E2F/CpG positive gene promoters.

Comparison of single positive gene promoters (identified by MACS and operation on genomic intervals; peaks/intervals in bed versus TSS ± 2 kbp, as described in Materials and Methods) using a Venn diagram allowed the identification of regions enriched in PARP1/BRG1/H3K27ac/E2F/CpG in all possible combinations. E2F promoters represent the joint list for E2F1 and E2F4. In brief, “only E2F” means that the listed promoters were characterized only by the presence of the E2F binding site, while E2F/CpG showed that the promoters shared both features. E2F/CpG-positive promoters were then searched using Venn diagrams for PARP1, BRG1, and H3K27ac signatures.

Table 3. Full list of gene enrichment analysis for PARP1/BRG1/H3K27ac/E2F/CpG-positive promoters.

Gene ontology tested in AmiGO2 (binomial test with FDR correction) showed a long list of intracellular processes controlled by genes characterized by the presence of CpG islands, E2F binding motifs, and high nucleosome acetylation at promoters, which are simultaneously enriched in PARP1 and BRG1.

Table 4. Differential expression of proliferation and DNA repair genes in DCIS, MCF7 cells, and MDA-MB-231 cells versus normal breast tissue.

Quantitative changes in expression levels between experimental groups were analyzed by Cuffdiff (using cufflinks effective length correction, library normalization method. The geometric dispersion estimation method had a pooled false discovery rate of 0.05 and a minimum alignment count of 10) and revealed mostly over-expression of genes involved in cell division and removal of DNA lesions.

Table 5. Statistical analysis of data.

Data are shown as mean ± standard deviation of the mean (SEM). Student’s t-test was used to determine statistically significant differences between two means (marked with * when $p < 0.05$, ** when $p < 0.01$, and *** when $p < 0.001$), while one-way analysis of variance (ANOVA) was carried out in GraphPad Prism 5 to compare the means between several groups (marked with * when $p < 0.05$, ** when $p < 0.01$, and *** when $p < 0.001$)
